# Study of USH1 Splicing Variants through Minigenes and Transcript Analysis from Nasal Epithelial Cells

**DOI:** 10.1371/journal.pone.0057506

**Published:** 2013-02-25

**Authors:** María José Aparisi, Gema García-García, Elena Aller, María Dolores Sequedo, Cristina Martínez-Fernández de la Cámara, Regina Rodrigo, Miguel Armengot, Julio Cortijo, Javier Milara, Manuel Díaz-LLopis, Teresa Jaijo, José María Millán

**Affiliations:** 1 Research Group on Sensorineural Diseases, Instituto de Investigación Sanitaria - La Fe, Valencia, Spain; 2 Biomedical Network Research Center for Rare Diseases, Valencia, Spain; 3 Rhinology Unit, General and University Hospital, Medical School, Valencia University, Valencia, Spain; 4 Research Foundation of the University General Hospital of Valencia, Valencia, Spain; 5 Biomedical Network Research Center for Respiratory Diseases, Valencia, Spain; 6 University of Valencia, Valencia, Spain; 7 Clinical Pharmacology Unit, University Clinic Hospital, Valencia, Spain; 8 Research Unit, University General Hospital Consortium, Valencia, Spain; 9 Department of Ophthalmology, La Fe University Hospital, Medical School, Valencia University, Valencia, Spain; 10 Genetics Unit, La Fe University Hospital, Valencia, Spain; International Centre for Genetic Engineering and Biotechnology, Italy

## Abstract

Usher syndrome type I (USH1) is an autosomal recessive disorder characterized by congenital profound deafness, vestibular areflexia and prepubertal retinitis pigmentosa. The first purpose of this study was to determine the pathologic nature of eighteen USH1 putative splicing variants found in our series and their effect in the splicing process by minigene assays. These variants were selected according to bioinformatic analysis. The second aim was to analyze the USH1 transcripts, obtained from nasal epithelial cells samples of our patients, in order to corroborate the observed effect of mutations by minigenes in patient’s tissues. The last objective was to evaluate the nasal ciliary beat frequency in patients with USH1 and compare it with control subjects. *In silico* analysis were performed using four bioinformatic programs: *NNSplice*, *Human Splicing Finder*, *NetGene2* and *Spliceview.* Afterward, minigenes based on the pSPL3 vector were used to investigate the implication of selected changes in the mRNA processing. To observe the effect of mutations in the patient’s tissues, RNA was extracted from nasal epithelial cells and RT-PCR analyses were performed. Four *MYO7A* (c.470G>A, c.1342_1343delAG, c.5856G>A and c.3652G>A), three *CDH23* (c.2289+1G>A, c.6049G>A and c.8722+1delG) and one *PCDH15* (c.3717+2dupTT) variants were observed to affect the splicing process by minigene assays and/or transcripts analysis obtained from nasal cells. Based on our results, minigenes are a good approach to determine the implication of identified variants in the mRNA processing, and the analysis of RNA obtained from nasal epithelial cells is an alternative method to discriminate neutral Usher variants from those with a pathogenic effect on the splicing process. In addition, we could observe that the nasal ciliated epithelium of USH1 patients shows a lower ciliary beat frequency than control subjects.

## Introduction

Usher syndrome (USH) is an autosomal recessive disorder characterized by sensorineural hearing loss, retinitis pigmentosa (RP) and variable vestibular dysfunction. USH is clinically and genetically heterogeneous and is the most common form of deaf-blindness of genetic origin, representing 50% of cases [1]. This disease shows a prevalence of 3.2–6.2/100000 [2], [3], [4].

Three clinical types of USH (types I, II and III; USH1, USH2 and USH3) are recognized, mainly on the basis of the severity and progression of hearing loss, the age of onset of RP and the presence of vestibular dysfunction [5].

Usher syndrome type I (USH1) is the most severe form of the disease and it is characterized by congenital profound deafness, vestibular areflexia and prepubertal onset of retinitis pigmentosa. To date, nine loci (USH1B-USH1K) have been mapped and six genes have been identified: *MYO7A* (USH1B): MIM#276903; *USH1C* (USH1C): MIM# 605242; *CDH23* (USH1D): MIM# 605516; *PCDH15* (USH1F): MIM# 605514; *USH1G* (USH1G): MIM# 607696; and *CIB2* (USH1J): MIM# 605564 [reviewed in 6], [7], [8].

Many mutations in *MYO7A*, *USH1C*, *CDH23*, *PCDH15* and *USH1G* have been identified by several screenings performed in USH1 patients (http://grenada.lumc.nl/LOVD2/Usher_montpellier/). The consequences of missense, silent and intronic changes many times are unknown and additional studies are needed to know the pathogenicity of these variants.

The use of minigene assays has been shown to be a useful approach to determine the effect of these variants on the splicing process, when genes present a restricted expression profile (photoreceptors and inner hair cells, in the case of USH) and human specific tissue samples are difficult to obtain [9].

Cohn et al. [10] demonstrated the presence of eight Usher proteins in nasal ciliated epithelium using immunochemistry with fluorescent antibodies. Subsequently, Vaché et al. [11] provided evidence that splicing mutations occurring in most USH genes can be identified through *ex vivo* analysis of mRNA from nasal epithelial cells.

On the other hand, the cilium in photoreceptors appears ultrastructurally and molecularly very similar to the nasal ciliated epithelium. The cilia are distributed around the human body and it has been reported that an abnormality in ciliary function may be linked to the nasal cilia abnormalities, as well as to the retinal degeneration [12]. There is evidence that immotiles nasal cilial can be associated with USH1 [13].

In our cohort of patients, we identified different pathogenic variants and some putative splicing mutations in USH1 genes [14], [15], [16], [17], [18].

The first purpose of the present work was to determine the pathogenic nature of selected variants and their effect in the splicing process by minigene assays. The second aim was to analyze the USH1 transcripts, obtained from the nasal epithelium cells of our patients, in order to corroborate the observed effect of mutations by minigenes in patient’s tissues. The third goal of this study was to evaluate the nasal ciliary beat frequency in eight USH1 patients and compare it with thirty control subjects.

## Materials and Methods

### Ethics Statement

This study was approved by the ethic committees of the Instituto de Investigación Sanitaria IIS-La Fe (Valencia, Spain) and the General and University Hospital (Valencia, Spain). Written consent was obtained from all subjects. Clinical investigation was conducted according to the principles expressed in the Declaration of Helsinki.

### Selection of Variants

The first selection of sequence variations for functional studies was carried out on the following criteria: variants found in a homozygous state or in trans with a disease-causing mutation, cosegregation with the disease, not found in 200 control chromosomes or sequence variation located in exon or introns that may affect the mRNA processing. All changes have been characterized in our cohort of patients.

### Computational Analysis of Splicing Variants

To analyze the effect of variants in the splice prediction and the recognition of donor and acceptor sites, *in silico* analyses were performed. Four programs were used: Neural Network SPLICE (*NNSplice)* 0.9 from the Berkeley Drosophila Genome Project (available at http://www.fruitfly.org/seq_tools/splice.html), *Human Splicing Finder (HSF)*-Version 2.4 (available at http://www.umd.be/HSF/), *NetGene2* (available at http://www.cbs.dtu.dk/service/NetGene2/) and *Spliceview* (available at http://zeus2.itb.cnr.it/~webgene/wwwspliceview_ex.html).Score-values were calculated taking into account the bioinformatic predictions, from 1 to 4, depending on how many of the four programs have predicted changes on the splice sites.

### Minigene Constructions and Expression

Minigene constructions based on the pSPL3 exon trapping vector (kindly provided by Dr. I Botillo and Dr. S. Tuffery-Giraud) were used to investigate the implication of selected variants on the mRNA processing. These variants were selected based on the bioinformatic predictions.

For these putative splicing variants, the exon and intronic flanking sequences were amplified from the patient’s DNA, using the High Fidelity Phusion polymerase (Finnzymes, Espoo, Finland) with primers detailed in Table 1. Amplicons were inserted between the XhoI/NheI restriction sites, using T4 DNA ligase (Invitrogen Corporation, Carlsbad, CA). We only could obtain the wild type (WT) insert for the variant c.3717+2dupTT (*PCDH15*) and the mutant insert for changes c.3652G>A and c.5581C>T (*MYO7A*), c.1086-12G>A (*USH1C*), c.8722+1delG (*CDH23*) and c.1304_1305insC, c.2868+5G>A and c.1737C>G (*PCDH15*). The corresponding WT or mutant insert was obtained by site-directed mutagenesis using primers detailed in Table 2. Minigene constructions were confirmed by direct sequencing in both directions with BigDye Terminator 3.1 cycle sequencing kit from Applied Biosystems (Carlsbad, USA). Sequence reactions were analyzed on a capillary ABI 3500xl Genetic Analyzer (Applied Biosystems) according to the manufacturer’s instructions. Afterwards, minigenes were transfected into COS-7 cells as described before by Jaijo et al. [19]. RNA extraction and RT-PCR analysis was performed as previously described [20], [21]. All experiments were performed in duplicate.

**Table 1:** Primers used to amplify the specific insert.

Sequence variant	Primer	Sequence 5′->3′	Size (bp)
c.6_9dup (p.L4DfsX39)*MYO7A*	L4DfsX39_D_XhoI	**AAGAAT** *CTCGAG* AGTGGCTGAGAGAAGAATTC	563
	L4DfsX39_R_NheI	**AAGAAT** *GCTAGC* GGATCCAGAGATGTGTGTAA	
c.470G>A (p.S157N) *MYO7A*	S157N_D_XhoI	**AAGAAT** *CTCGAG* AGACTCCACCTCCCTCTTCA	668
	S157N_R_NheI	**AAGAAT** *GCTAGC* GCTAATGTGAGCTTTGGTAGC	
c.640G>A (p.G214R) *MYO7A*c.721C>G (p.R241G) *MYO7A*	G214R & R241G_D_XhoI	**AAGAAT** *CTCGAG* GAAGGTGAAGGAGAGTGCG	703
	G214R & R241G_R_NheI	**AAGAAT** *GCTAGC* TTCATGGTGGGATTTCCAGC	
c.1097T>C (p.L366P)*MYO7A*	L366P_D_XhoI	**AAGAAT** *CTCGAG* TGACATGCTGGAGGGAGTTA	608
	L366P_R_NheI	**AAGAAT** *GCTAGC* CTGACCAAAGCAGGCCAAAA	
c.1342_1343delAG (p.S448LfsX2) *MYO7A*	S448LfsX2_D_XhoI	**AAGAAT** *CTCGAG* TCTCCAGGCAGAGGGAACAG	524
	S448LfsX2_R_NheI	**AAGAAT** *GCTAGC* AGCCAGGCTCAGCTGACCTT	
c.3508G>A (p.E1170K) *MYO7A*	E1170K_D_XhoI	**AAGAAT** *CTCGAG* GAGGTGCTTTATGCCCGATG	545
	E1170K_R_NheI	**AAGAAT** *GCTAGC* GAGTGTGGGGAAAAGAGGTG	
c.3652G>A (p.G1218R) *MYO7A*	G1218R_D_XhoI	**AAGAAT** *CTCGAG* ATAGATGGTGGAGCTGAGAG	370
	G1218R_R_NheI	**AAGAAT** *GCTAGC* GCACTGGACACACACACACA	
c.5581C>T (p.R1861X) *MYO7A*	R1861X_D_XhoI	**AAGAAT** *CTCGAG* ACTAGTTGCATCTGGCTGTC	543
	R1861X_R_NheI	**AAGAAT** *GCTAGC* ATCCTGAGCAGCTGCAGGAT	
c.1086-12G>A *USH1C*	USH1C_D_XhoI	**AAGAAT** *CTCGAG* ACAAGCTCGGGGACTTTGCT	438
	USH1C_R_NheI	**AAGAAT** *GCTAGC* ATGGTCTCTGACCCACAGCT	
c.2289+1G>A *CDH23*	c.2289_D_XhoI	**AAGAAT** *CTCGAG* CAAATATTTCCTCCTCATGG	636
	c.2289_R_NheI	**AAGAAT** *GCTAGC* GCAGGAGAGAAAGTATTTCA	
c.6049G>A (p.G2017S) *CDH23*	G2017_D_XhoI	**AAGAAT** *CTCGAG* CTTGCTCACCACTCTCATGT	594
	G2017_R_NheI	**AAGAAT** *GCTAGC* GTATCGTCTCCAAGCCTCTT	
c.8722+1delG *CDH23*	c.8722_D_XhoI	**AAGAAT** *CTCGAG* TTCTGGGCAACACTCCGTGA	1226
	c.8722_R_NheI	**AAGAAT** *GCTAGC* GGATGGACGGATAACTAATGG	
c.1304_1305insC *PCDH15*	c.1304_D_XhoI	**AAGAAT** *CTCGAG* CAGAAGACTGAAGCAATTAAGCC	647
	c.1304_R_NheI	**AAGAAT** *GCTAGC* ATATACTGAATATCTGCAAGCTG	
c.2868+5G>A *PCDH15*	c.2868_D_XhoI	**AAGAAT** *CTCGAG* ATTCCAGTGGAACGGCCTTC	533
	c.2868_R_NheI	**AAGAAT** *GCTAGC* TATGCTCTGTACCTGTGGATG	
c.3717+2dupTT *PCDH15*	c.3717_D_XhoI	**AAGAAT** *CTCGAG* CAGAATTATACCTATGTCCC	635
	c.3717_R_NheI	**AAGAAT** *GCTAGC* AGTGACTAACAATCTGAGTG	
c.521A>G (p.N174S) *PCDH15*	N174S_D_XhoI	**AAGAAT** *CTCGAG* TTCTTCGCCCATAGCAAGA	605
	N174S_R_NheI	**AAGAAT** *GCTAGC* TGAGGCATATTATACCTATG	
c.1737C>G (p.Y579X) *PCDH15*	Y579X_D_XhoI	**AAGAAT** *CTCGAG* AGTATTGTAACAGGACACAG	596
	Y579X_R_NheI	**AAGAAT** *GCTAGC* CCTCTGATATTGTCCTCTTC	

Tails added at the beginning of the primer are indicated in bold.

Enzyme restriction sites used in this study are indicated in italics.

**Table 2:** Primers used for the site-directed mutagenesis.

Sequence variants	Primer	Sequence 5′->3′
c.3717+2dupTT *PCDH15* WT	c.3717-D-WT	GCAAAGCCGATGTACTCGT-AAGTAGATAAAACTTCAGG
	c.3717-R-WT	CCTGAAGTTTTATCTACTT-ACGAGTACATCGGCTTTGC
c.5581C>T (p.R1861X) *MYO7A* MUT	R1861X-D-MUT	GCTTCCTGCAGTCC**C**GAAAGCACTGCCCA
	R1861X-R-MUT	TGGGCAGTGCTTTC**G**GGACTGCAGGAAGC
c.3652G>A (p.G1218R) *MYO7A* MUT	G1218R-D-MUT	AACTTCATCCAC**A**GGGGCCCGCCCG
	G1218R-R-MUT	CGGGCGGGCCCC**T**GTGGATGAAGTT
c.1086-12G>A *USH1C* MUT	c.1086-D-MUT	CCAGTAACAGGCATGGGG**A**TCTCATTTTAGGATTGTAG
	c.1086-R-MUT	CTACAATCCTAAAATGAGA**T**CCCCATGCCTGTTACTGG
c.8722+1delG *CDH23* MUT	c.8722-D-MUT	GGTCTTCACCATGG-TAGGGCCTGGCAGC
	c.8722-R-MUT	GCTGCCAGGCCCTA-CCATGGTGAAGACC
c.1304_1305insC *PCDH15* MUT	c.1304-D-MUT	GTAGCTCTGGACAAGGACATAGAAGA**C**TGTAAGTTAAATACATATTTTGC
	c.1304-R-MUT	GCAAAATATGTATTTAACTTACA**G**TCTTCTATGTCCTTGTCCAGAGCTAC
c.2868+5G>A *PCDH15* MUT	c.2868-D-MUT	GAAGATGCAGACCCTCCTGTAA**A**TAGAAGGCATTGATTAATATT
	c.2868-R-MUT	AATATTAATCAATGCCTTCTA**T**TTACAGGAGGGTCTGCATCTTC
c.1737C>G (p.Y579X) *PCDH15* MUT	Y579X-D-MUT	TGATAGTCGGGCGGACTTA**G**GCACTCACGG
	Y579X-R-MUT	CCGTGAGTGC**C**TAAGTCCGCCCGACTATCA

The nucleotides that have been modified are indicated in bold.

The positions of deleted nucleotides are represented with (−).

### Nasal Epithelial Cells Samples from Controls and Patients

A total of thirty-eight fresh nasal samples were obtained from eight USH1 patients, five patients with mutations in *MYO7A* gene and three patients with mutations in the *CDH23* gene, and thirty control subjects. The nasal samples were obtained from the middle nasal concha using curettage without local anesthesia, in a period during which acute infection was absent [12], in the ENT service of the Hospital General de Valencia, Spain.

### RNA and Sequence Analysis

RNA extraction from nasal epithelial cells and RT-PCR analysis was performed as previously described [11]. cDNA was used as template in nested PCR reactions with specific primers in order to amplify the regions containing the mutation (Table 3). PCR products were tested on 1.5% agarose gel and purified by ExoSAP. PCR products were sequenced and analyzed. All experiments were performed in duplicate.

**Table 3:** Primers used to amplify fragments of *MYO7A* and *CDH23* cDNAs from nasal cells.

Gene	Exons	Primers	Sequence 5′->3′	Size(bp)
*MYO7A*	1–5	MYO_EXP_1-5-EXT-D	AGAGACAAGAGACACACACA	628
		MYO_EXP_1-5-EXT-R	CTTCTTGTTGGTATACTGGC	
		MYO_EXP_1-5-INT-D	TAGAACGAGACTTGGAGCCA	490
		MYO_EXP_1-5-INT-R	TTCACAGCCACCAGGATGGA	
	3–9	MYO_EXP_3-9-EXT-D	ACCATGTGTGGATGGACCTG	937
		MYO_EXP_3-9-EXT-R	CTTCGAGATCTCCCAGTTCT	
		MYO_EXP_3-9-INT-D	ACTCTGGGCAGGTCCAGGT	806
		MYO_EXP_3-9-INT-R	TGTTGGCGTACTCCTGGCT	
	8–14	MYO_EXP_8-14-EXT-D	TGTTCTACTGCATGCTGGAG	906
		MYO_EXP_8-14-EXT-R	CCTGCAAAATGGTTGATGCC	
		MYO_EXP_8-14-INT-D	AAGAAGAAGCTGGGTTGGG	811
		MYO_EXP_8-14-INT-R	TTGGCGTTGAGCTTGTGCT	
	25–33	MYO_EXP_25-33-EXT-D	ATGAGACCCTGGGCAAGAAG	1136
		*MYO_EXP_25-33-EXT-R	CTTCTGGGCATCAGTTCTCC	
		MYO_EXP_25-33-INT-D	AGGGCCAGAAGAAGAGCAGT	924
		MYO_EXP_25-33-INT-R	CGCTCCAGGATCATCTCAGA	
	38–45	MYO_EXP_38-45-EXT-D	TCCTATGACTACTTCAGGCC	1010
		MYO_EXP_38-45-EXT-R	GGGGAAGTAGGACTTGTCCT	
		MYO_EXP_38-45-INT-D	TCAAGCAGGCGCTGCTCAAGA	835
		MYO_EXP_38-45-INT-R	CGTGCACTTGTGGTAGCCTC	
*CDH23*	18–24	CDH_EXP_18-24-EXT-D	ATGAACAGATATCCAATGGGC	790
		CDH_EXP_18-24-EXT-R	GGTTCTGAAAGGTGGGGTCA	
		CDH_EXP_18-24-INT-D	AATGACAACCCTCCCACCTT	627
		CDH_EXP_18-24-INT-R	CACTGCTGGTGGCTTTCAGA	
	43–49	CDH_EXP_43-49-EXT-D	CATCAACGACAACGACCCTGT	1084
		CDH_EXP_43-49-EXT-R	GGTTGAGGTCGTGGTCAATG	
		CDH_EXP_43-49-INT-D	TCTGTGAAGGACAACCCGGA	708
		CDH_EXP_43-49-INT-R	GAATGGTGACAATGGCTGTG	
	57–63	CDH_EXP_57-63-EXT-D	CTCATCTTGGTGGCCAGCGAC	1015
		CDH_EXP_57-63-EXT-R	CCGGCAGCCGGACAGAGAT	
		CDH_EXP_57-63-INT-D	TTCATCGTCAAGGCCTCCAG	713
		CDH_EXP_57-63-INT-R	TAGCTGCTCCTTGTTCTCA	

* The primer MYO_EXP_25-33-EXT-R was designed by Vaché et al. [11].

### Nomenclature of Variants

The sequences obtained were compared with the consensus sequence NM 000260.3 for *MYO7A,* NM 153676.2 for *USH1C*, NM 022124.3 for *CDH23* and NM 033056.3 for *PCDH15.*

For the protein nomenclature, we used the Mutalyzer 2.0 beta-21 program (available at https://mutalyzer.nl/).

### Nasal Ciliary Beat Frequency

Ciliary beat frequency was measured as described by Armengot et al. [12] in eight USH1 patients and thirty healthy subjects. The beat pattern was observed using high-resolution digital high-speed video imaging. Values were expressed in *Hertz* (Hz) as the mean ± standard error of mean.

### Statistical Analysis

Ciliary beat frequencies were analyzed by Kruskal-Wallis test followed by Dunns post-hoc test. When only two groups were compared, *Mann-Whitney* U test was used. Significance levels were set at p<0.05. Data were analyzed with statistical analysis software GraphPad Prism (Version 5.01, San Diego, USA).

## Results

### Computational Analysis

One hundred and eight variants identified in the USH1 genes were studied with bioinformatic tools (*SpliceView, NNSplice, NetGene2* and *HSF*) in order to analyze the effect of these variants in the splice prediction and the recognition of donor and acceptor sites. Nine *MYO7A*, three *CDH23*, five *PCDH15* and one *USH1C* variants were predicted to alter the splicing mechanism creating or eliminating donor/acceptor splice sites, see table 4. Considering the four programs (*Splice View, NNSplice, NetGene2* and *HSF*) the score-values were calculated. Eight out of the eighteen variants showed the highest score (4), five of them showed a score of 3, two of the variants were observed to show a score of 2 and the three remaining changes showed a score of 1.

**Table 4:** Results from four different bioinformatic programs used to predict the effect on the splicing process.

Sequence variants	Type ofsplicesite	*NetGene2*	*HSF*	*NNSplice*	*Splice View*	Score
**c.6_9dup (p.L4DfsX39)** ***MYO7A*** **** [**14**]	Acceptor	Score for acceptor siteincreases from 77 to 82	The WT consensus sequenceis not recognized	One donor site is not recognized	New acceptor sites are created and other acceptor sites are not recognized	3
**c.470G>A (p.S157N)** ***MYO7A*** **** [**14**]	Donor	Score for the main donorsite decreases from 93 to 60	Score for donor site decreasesand a new acceptor site iscreated	The main donor site isnot recognized	The main donor site isnot recognized	4
**c.640G>A (p.G214R)** ***MYO7A*** **** [**22**]	Acceptor	Neutral	The WT consensus sequenceis not recognized	A new acceptor site is created	Neutral	1
**c.721C>G (p.R241G)** ***MYO7A*** **** [**15**]	Donor	Three new donor siteare created	A new acceptor siteis created	Score for the main acceptor site decreases from 81 to 59	A new donor site is created	4
**c.1097T>C (p.L366P)** ***MYO7A*** **** [**15**]	Acceptor	Score for the main acceptorsite decreases from 83 to 77	Score for the acceptorsite decreases	A new acceptor site is created	Neutral	3
**c.1342_1343delAG (p.S448LfsX2)** ***MYO7A*** **** [**14**]	Donor	The main donor site is notrecognized	The main donor site and theacceptor site arenot recognized	The main donor site isnot recognized	The main donor site isnot recognized	4
**c.3508G>A (p.E1170K)** ***MYO7A*** **** [**23**]	Acceptor	Score for the main acceptorsite decreases from 85 to 77	Neutral	Neutral	Neutral	1
**c.3652G>A (p.G1218R)** ***MYO7A*** **** [**15**]	Acceptor	A new acceptor siteis created	A new acceptor siteis created	A new acceptor site is created	A new acceptor site is created	4
**c.5581C>T (p.R1861X) ** ***MYO7A*** **** [**22**]	Acceptor	Score for the main acceptorsite decreases from 77 to 72	Neutral	Neutral	Neutral	1
**c.1086-12G>A** ***USH1C*** **** [**17**]	Acceptor	A new acceptor siteis created	Score for acceptor sitedecreases	Score for the main acceptor site decreases from 48 to 45	The main acceptor site isnot recognized	4
**c.2289+1G>A** ***CDH23*** **** [**24**]	Donor	The main donor site isnot recognized	The main donor site is notrecognized	The main donor site isnot recognized	The main donor site isnot recognized	4
**c.6049G>A (p.G2017S)** ***CDH23*** **** [**25**]	Donor	Neutral	The main donor site is notrecognized	The main donor site isnot recognized	Score for the maindonor site decreasesfrom 89 to 84	3
**c.8722+1delG** ***CDH23*** **** [**16**]	Donor	Neutral	The main donor site is notrecognized	The main donor site isnot recognized	The main donor site isnot recognized	3
**c.521A>G (p.N174S)** ***PCDH15*** **** [**18**]	Acceptor	Score for the main acceptorsite decreases from 92 to 87	The main acceptor site isnot recognized	Neutral	Neutral	2
**c.1304_1305insC (p.T436YfsX12)** ***PCDH15*** **** [**18**]	Donor	Score for the main donorsite decreases from 63 to 51	The WT consensus sequenceis not recognized	Score for the maindonor site decreasesfrom 99 to 94	Score for the main donor site decreases from 81 to 79	3
**c.1737C>G (p.Y579X)** ***PCDH15*** **** [**18**]	Donor	Score for the main donorsite decreases from 52 to 37	The main donor siteis not recognized	Neutral	Neutral	2
**c.2868+5G>A** ***PCDH15*** **** [**18**]	Donor	The main donor site is notrecognized and a newdonor site is created	The main donor siteis not recognized.	The main donor site isnot recognized.	The main donor site isnot recognized.	4
**c.3717+2dupTT ** ***PCDH15*** **Present study**	Donor	The main donor site isnot recognized	The main donor siteis not recognized	The main donor site isnot recognized	The main donor site isnot recognized	4

### Minigene Constructions

In order to confirm the bioinformatic predictions, minigenes were constructed and the different splicing products were analyzed. The minigene assays showed that only seven of them were affecting the mRNA processing: three *MYO7A* variants, c.470G>A, c.1342_1343delAG and c.3652G>A, three *CDH23* changes, c.2289+1G>A, c.6049G>A and c.8722+1delG, and one *PCDH15* variant, c.3717+2dupTT, see Table 5.

**Table 5:** Effects of USH1 variants on splicing**.**

Putative splicing variants	Scoreaccording tobioinformatictools	Effect on RNA level according to minigene results	Effect on protein level according to minigene results	Effect on RNA level according to nasal cells results	Effect on protein level according to nasal cells results
**c.6_9dup (p.L4DfsX39) ** ***MYO7A***	3	No effect on splicing	p.L4DfsX39	No effect on splicing	p.L4DfsX39
**c.470G>A (p.S157N) ** ***MYO7A***	4	Exon skipping	p.T96WfsX29	–	–
**c.640G>A (p.G214R) ** ***MYO7A***	1	No effect on splicing	p.G214R	No effect on splicing	p.G214R
**c.721C>G (p.R241G) ** ***MYO7A***	4	No effect on splicing	p.R241G	–	–
**c.1097T>C (p.L366P) ** ***MYO7A***	3	No effect on splicing	p.L366P	–	–
**c.1342_1343delAG (p.S448LfsX2) ** ***MYO7A***	4	The mutation removes the main donorsite and creates a new donor splice-siteleading to a deletion of 24bp	p.N443_E450del	–	–
**c.3508G>A (p.E1170K) ** ***MYO7A***	1	No effect on splicing	p.E1170K	No effect on splicing	p.E1170K
**c.3652G>A (p.G1218R) ** ***MYO7A***	4	The mutation does not recognize the acceptorsite and a new acceptor splice-site is createdleading to a deletion of 103-bp in the 5′end of exon 29	p.Y1211AfsX18	–	–
**c.5581C>T (p.R1861X) ** ***MYO7A***	1	No effect on splicing	p.R1861X	No effect on splicing	p.R1861X
* **c.5856G>A (p.K1952K) ** ***MYO7A***	4	Exon skipping	p.A1915_K1952del	Exon skipping	p.A1915_K1952del
**c.1086-12G>A ** ***USH1C***	4	No effect on splicing	Neutral	–	–
**c.2289+1G>A ** ***CDH23***	4	This variant does not recognize the main donorsite and a new donor splice-site is createdinserting the first 149 nucleotides of intron21+ Exon skipping	p.N765SfsX35+ p.E727KfsX9	Transcript with an insertionof the first 149 nucleotides of intron 21 plus the last 54 nucleotides of the same intron	p.N765SfsX35
**c.6049G>A (p.G2017S) ** ***CDH23***	3	Exon skipping	p.T1976_G2017del	Probable NMD	-
**c.8722+1delG ** ***CDH23***	3	Deletion of the last nucleotide of exon 60	p.S2909AfsX43	Deletion of the lastnucleotide of exon 60	p.S2909AfsX43
**c.521A>G (p.N174S) ** ***PCDH15***	2	No effect on splicing	p.N174S	–	–
**c.1304_1305insC (p.T436YfsX12) ** ***PCDH15***	3	No effect on splicing	p.T436YfsX12	–	–
**c.1737C>G (p.Y579X) ** ***PCDH15***	2	No effect on splicing	p.Y579X	–	–
**c.2868+5G>A ** ***PCDH15***	4	No effect on splicing	Neutral	–	–
**c.3717+2dupTT ** ***PCDH15***	4	c.3717+2dupTT does not recognize the maindonor site and creates a new donor splice-sitethat includes the first 52 nucleotides ofintron 27+ Exon skipping	p.V1242RfsX2+ p.A1168_L1239del	–	–

Not performed in this study are indicated with (−).

NMD: Nonsense mediated decay.

* c.5856G>A (p.K1952K, *MYO7A*) was previously analyzed by Jaijo et al. [26].

#### c.470G>A (p.S157N, *MYO7A*)

**Figure 1:**
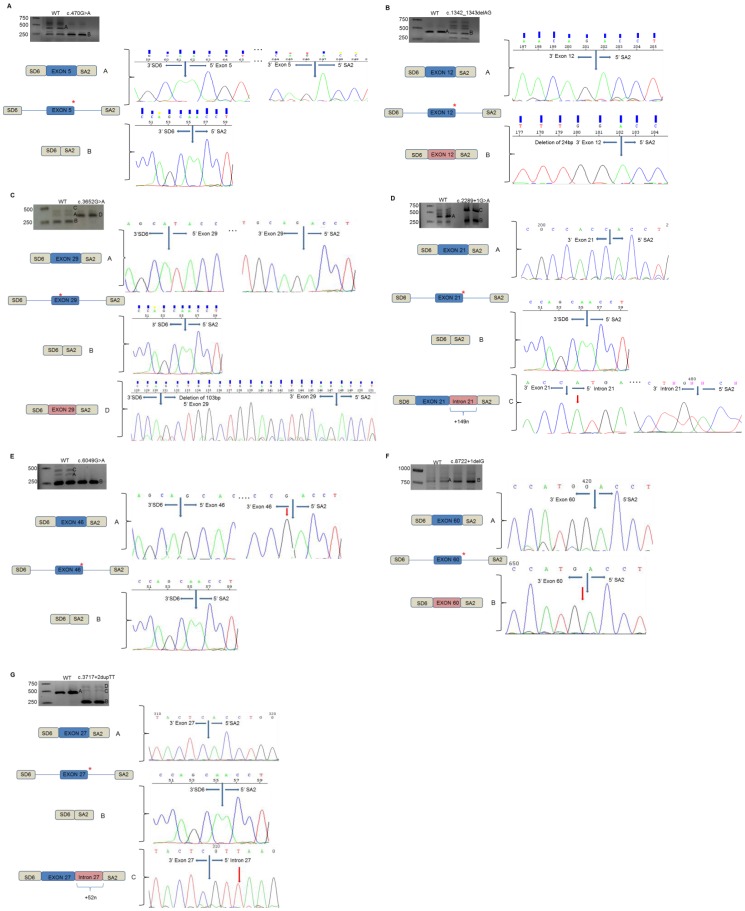
In vitro splicing assays for the seven splicing mutations identified in the USH1 genes. Gel electrophoresis shows the different splicing processes for WT minigene and mutants constructions. COS-7 cell transfection experiments were performed in duplicate. **A. c.470G>A (p.S157N,** ***MYO7A*** **).** Band A is the correct transcript of exon 5 (*MYO7A*). Band B is the skipping of involved exon. **B. c.1342_1343delAG (p.S448LfsX2,** ***MYO7A*** **).** Band A is the correct transcript corresponding to the exon 12 (*MYO7A*). Band B is the skipping of exon 12. **C. c.3652G>A (p.G1218R** ***MYO7A*** **).** Band A is the correct transcript of exon 29 (*MYO7A*). The band B is the exon skipping. Band C is the heteroduplex formation from band A and band B. Band D is the aberrant splicing process that include the deletion of 103-bp of 5′end of exon 29. **D. c.2289+1G>A (** ***CDH23*** **).** Band A is the normal transcript of exon 21 (*CDH23*). Band B is the skipping of exon 21. Band C is the aberrant splicing process that includes the first 149 nucleotides of intron 21. **E. c.6049G>A (p.G2017S,** ***CDH23*** **).** Band A is the correct transcript of exon 46 (*CDH23*). Band B is the skipping of exon 46. Band C is the heteroduplex formation from the band A and B. **F. c.8722+1delG (** ***CDH23*** **).** Band A is the correct splicing process of exon 60 (*CDH23*). Band B is the abnormal splicing process of exon 60 that shows a deletion of the last nucleotide (G) of the involved exon. **G. c.3717+2dupTT (** ***PCDH15*** **).** Band A is the correct transcript of exon 27 (*PCDH15*). Band B is the skipping of the involved exon. Band C is the transcript corresponding to the new donor splice site from exon 27 plus the first 52 nucleotides of the intron 27. Band D is the heteroduplex formation from the band B and the band D.

The minigene assay showed that the processing of the WT minigene generated two main fragments: the correct processing (band A) and the exon skipping (band B). The mutant minigenes only showed one fragment corresponding to the skipping of exon 5 (band B) (Fig. 1A). If this mutant transcript was translated, it would produce a truncated protein of only 123 amino acids in length, p.T96WfsX29.

#### c.1342_1343delAG (p.S448LfsX2, *MYO7A*)

*In vitro* analyses confirmed that the WT transcript contained the correct exon (band A) but the c.1342_1343delAG construction generated several bands. We were only able to sequence band B that corresponded to the exon 12 with a partial deletion. The mutation removed the main donor site and created a new donor splice-site leading to a deletion of 24-bp (Fig. 1B). Thus, the new myosin VIIA protein generated, if this aberrant transcript was translated, it would be of 2207 amino acids in length, p.N443_E450del.

#### c.3652G>A (p.G1218R, *MYO7A*)

*In vitro* experiments showed that WT minigene generated two different transcripts: one of them corresponded to the correct transcript (band A) and the smallest and strongest transcript was the skipping of exon 29 (band B). A third band (band C) was also observed corresponding to the heteroduplex formation from the two obtained transcripts. Mutant minigene only showed one transcript corresponding to the aberrant splicing process (band D). This mutation avoided the recognition of the main acceptor site and created a new acceptor splice-site leading to a deletion of 103-bp in the 5′ end of exon 29 (Fig. 1C). If the c.3652G>A transcript was translated, it would generate a new truncated protein of 1227 amino acids in length, p.Y1211AfsX18.

#### c.2289+1G>A (*CDH23*)

The WT minigene generated a strong band corresponding to the correct transcript (band A) and a thin band corresponding to the exon skipping (band B). The mutant minigene showed two strong bands; one of them was the transcript with the exon 21 plus part of the intron 21 (band C) and the other band was the exon skipping (band B). This variant generated two different transcripts: one of them did not recognize the main donor site and created a new donor site including the first 149 nucleotides of intron 21 and the other transcript produced the skipping of exon 21. Thus, c.2289+1G>A would create two different proteins; p.N765SfsX35, of 798 amino acids in length, would correspond to the exon 21 plus 149 nucleotides of intron 21, and the other new truncated protein would be p.E727KfsX9, of 734 amino acids in length (Fig. 1D).

#### c.6049G>A (p.G2017S, *CDH23*)

Minigene assays revealed that the WT construction created two different transcripts: one of them corresponded to the correct transcript (band A) and the smallest transcript was the skipping of exon 46 (band B). A third band was also observed corresponding to the heteroduplex formation from the two smaller transcripts (band C). However, the c.6049G>A construction only showed one band corresponding to the skipping of exon 46 (band B) (Fig. 1E). If the transcript containing the c.6049G>A variant was translated, it would generate a protein of 3312 amino acids in length, p.T1976_G2017del.

#### c.8722+1delG (*CDH23*)

*In vitro* experiments showed that the WT construction generated one band (band A) corresponding to the correct transcript and the mutant construction showed a band with apparently the same size of the correct transcript (band B). However, when these fragments were sequenced, we could observe that the two fragments were different. The mutation produced the displacement of the donor splice site one nucleotide upstream, being the last nucleotide of the exon 60 processed as intron (Fig. 1F). If this mutant transcript was translated, it would be generating a new truncated protein of 2950 amino acids in length, p.S2909AfsX43.

#### c.3717+2dupTT (*PCDH15*)

In our series of patients, we identified a novel variant in intron 27 of the *PCDH15* gene in one Spanish USH1 family in homozygous state. We confirmed by minigene assays that the WT construction generated one correct fragment (band A). However, the c.3717+2dupTT minigene showed two different transcripts: the smallest and strongest transcript corresponded to the skipping of exon 27 (band B) and transcript containing exon 27 plus part of intron 27 (band C). A bigger band was observed corresponding to the heteroduplex from the two transcripts (band D). This novel mutation generated two different transcripts: the smallest and strongest transcript that corresponded to the skipping of involved exon 27 and a second transcript corresponding to the no recognition of the main donor splice site and the creation of a new donor site that includes the first 52 nucleotides of intron 27 (Fig. 1G). Therefore, the mutation would be creating two different proteins; one of them, corresponding to the skipping of exon 27, would create an in-frame deletion of 72 amino acids in length, p.A1168_L1239del, and a new truncated protein, corresponding to the exon 27 plus the first 52 nucleotides of intron 27, of 1241 amino acids in length, p.V1242RfsX2.

### Nasal Epithelial Cells

To carry out the second goal of the present study, we designed different primers to amplify particular fragments of the *MYO7A* and *CDH23* cDNAs from the nasal epithelium cells of patients and controls, in order to corroborate the observed effect of mutations by minigenes in the patient’s tissues. We only could obtain samples from subjects carriers of eight of the nineteen variants analyzed *in vitro* by minigenes. These samples were obtained from five USH1 patients and two family healthy carriers of the mutations c.6_9dup (RP-1481) and c.640G>A (RP-1546) in the *MYO7A* gene (Table 6).

**Table 6:** Genotypes of the five USH1 patients and the two family healthy carriers of USH1 mutations presented in this study.

Patient	Gene	Allele 1/Allele 2
RP-1481*	*MYO7A*	**c.6_9dup (p.L4DfsX39)** (exon2)/+
RP-1546*	*MYO7A*	**c.640G>A (p.G214R)** (exon7)/+
RP-115	*MYO7A*	**c.3508G>A (p.E1170K)** (exon 28)/c.3238A>T (p.K1080X) (exon 25)
RP-1479	*MYO7A*	**c.5581C>T (p.R1861X)** (exon 40)/**c.5581C>T (p.R1861X)** (exon 40)
RP-280	*MYO7A*	**c.5856G>A (p.K1952K)** (exon 42)/c.1190C>A (p.A397D) (exon 11)
RP-1534	*CDH23*	**c.2289+1G>A** (intron 21)/**c.6049G>A** (**p.G2017S)** (exon46)
RP-928	*CDH23*	**c.8722+1delG** (intron 60)/c.6511delC (exon48)

Family healthy carriers are indicated with an asterisk (*).

Only in four of the eight studied variants (c.5856G>A in the *MYO7A* gene, c.2289+1G>A, c.6049G>A and c.8722+1delG in the *CDH23* gene), we could observe an abnormal splicing process *ex vivo* (Table 5). In the remaining four samples both WT and mutant alleles were amplified showing that the presence of the mutations did not affect the splicing process.

#### c.5856G>A (p.K1952K, *MYO7A*)

**Figure 2:**
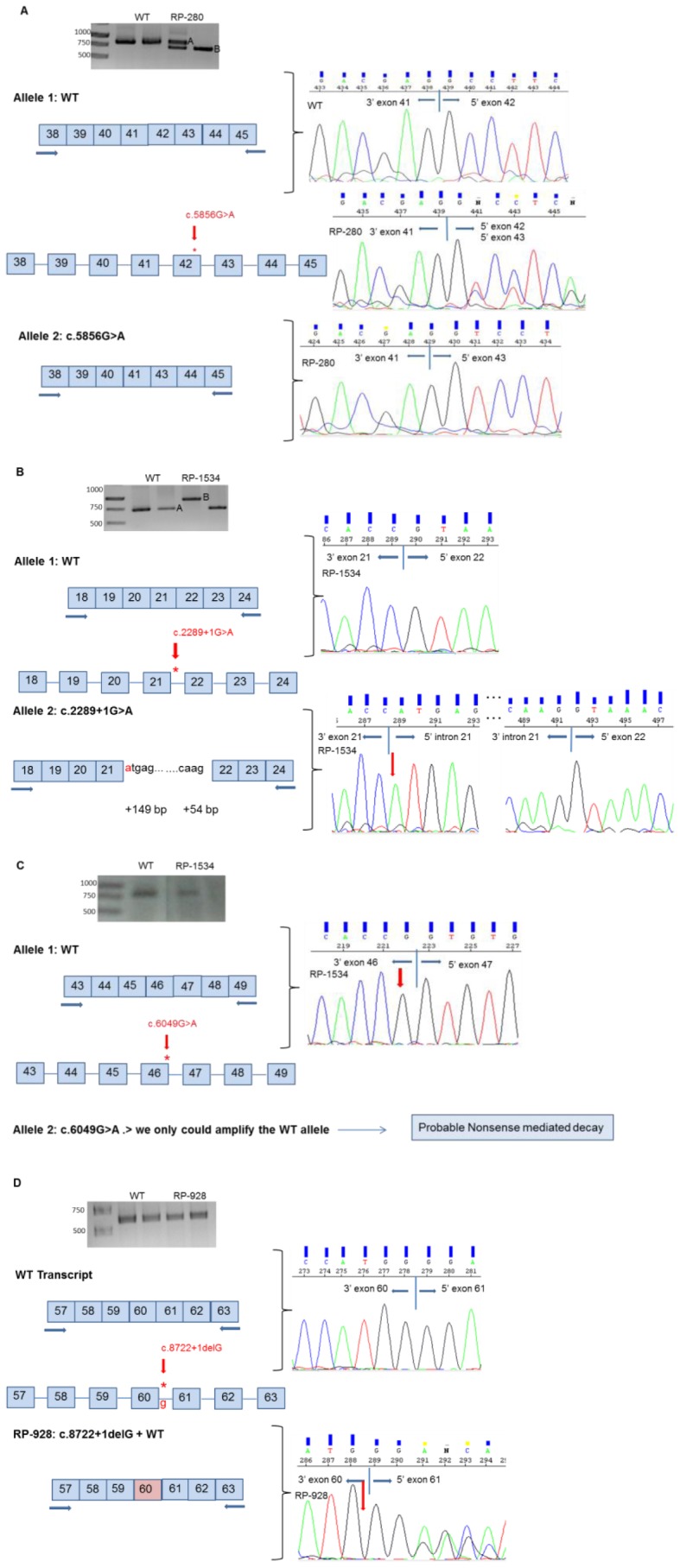
Transcript analysis of USH1 variants in nasal epithelial cells. Gel electrophoresis shows the RT-PCR products obtained for USH1 patients and control samples. Electropherograms of the transcripts obtained show the molecular characterization of the effect of the studied variants. All experiments were performed in duplicate. **A. c.5856G>A (p.K1952K,** ***MYO7A*** **).** Band A is the correct splicing process of the exons 38–45 of the *MYO7A* gene. Band B is the skipping of exon 42. RP-280 replicate 1 shows two transcripts corresponding to the WT allele (band A) and mutant allele (band B). RP-280 replicate 2 shows only the band B corresponding to the aberrant transcript. **B. c.2289+1G>A (** ***CDH23*** **).** Band A is the transcript corresponding to the normal RNA processing of the exons 18–24 of the *CDH23* gene. Band B is the aberrant transcript that includes the first 149 nucleotides and the last 54 nucleotides of intron 21**. C. c.6049G>A (p.G2017S,** ***CDH23*** **).** RP-1534 shows only the WT allele. **D. c.8722+1delG (** ***CDH23*** **).** RP-928 shows the heterozygous transcript from WT allele and the mutant allele. It presents a deletion of the last nucleotide (G) of exon 60 of *CDH23* gene.

Jaijo et al. [26] analyzed the c.5856G>A variant by minigene constructions. It showed the skipping of exon 42. The analysis of this change in the patient RP-280 replicate 1 revealed the presence of two transcripts: a fragment corresponding to the expected size, 835-bp also in the control sample (band A), and a fragment of 721-bp (band B). However, in the patient’s replicate 2, we only could amplify the smallest fragment. Sequencing of the products showed that the fragment of 835-bp corresponded to the normal allele. However, the product of 721-bp was the transcript without exon 42; if the transcript containing the p.K1952K variant was translated, it would create a new protein of 2177 amino acids in length, p.A1915_K1952del (Fig. 2A).

#### c.2289+1G>A *CDH23*

xamination of the RT-PCR product of the patient RP-1534 showed the presence of two transcripts, band A (observed in the replicate 2) and band B (observed in the replicate 1). However, in the control sample only one transcript was observed (band A). Sequencing of the band A showed the correct transcript of 627-bp. The patient’s replicate 1 amplified an abnormal transcript that included the first 149 nucleotides of intron 21 and the last 54 nucleotides of the same intron (band B) (Fig. 2B). Therefore, the mutation would be creating a new truncated protein of 798 amino acids in length, p.N765SfsX35.

#### c.6049G>A (p.G2017S, *CDH23*)

The patient RP-1534 also carried the p.G2017S. In this case, we only could amplify and sequence the normal allele (Fig. 2C).

#### c.8722+1delG (*CDH23*)

Analysis of the RT-PCR product of patient RP-928 showed only one band. However, sequencing clearly revealed the presence of two distinct transcripts: a normal spliced transcript and an abnormal transcript in which the splice site is modified causing the removal of one nucleotide (G) in the exons 60 and 61 boundary (Fig. 2D). Thus, if this mutant transcript was translated, the new protein would be of 2950 amino acids in length, p.S2909AfsX43.

### Ciliary Beat Frequency

**Figure 3:**
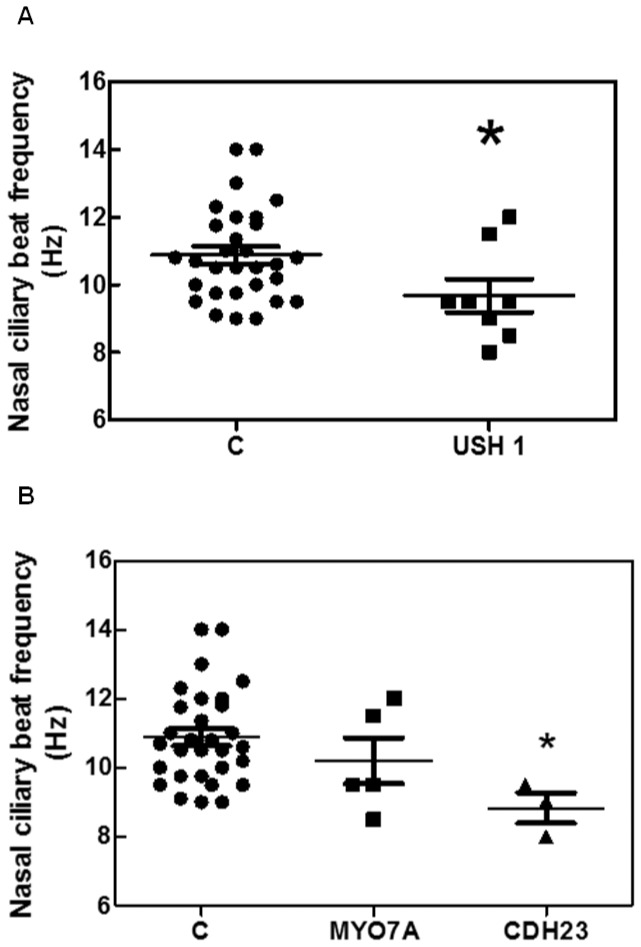
Nasal ciliary beat frequency in five *MYO7A* and three *CDH23* patients, and thirty controls. **A**. Comparison between USH1 group and Control group. The nasal ciliary beat frequency was significantly different between these groups (Mann Whitney Test, p  = 0.031). **B**. Comparison between the *MYO7A* group and *CDH23* group, *MYO7A* and Control group and *CDH23* group and Control group. The nasal ciliary beat frequency was significantly different between *CDH23* group and Control group (P<0.05).

The ciliary beat frequencies obtained from each patient and control were summarized in Table 7. Ciliary beat pattern was normal in all the patients. We analyzed whether USH1 patients showed differences in ciliary beat frequency compared to controls. As shown in Fig. 3A, the ciliary beat frequency was significantly reduced in these patients (9.68±0.49 Hz, Mann Whitney U test, p  = 0.031) compared to controls (10.88±0.25 Hz).

**Table 7:** Nasal ciliary beat frequency of USH1 patients and controls.

*MYO7A* patients	Nasal Ciliary Beat Frequency (Hz)
1	8.5
2	12
3	11.5
4	9.5
5	9.5
*CDH23* patients	Nasal Ciliary Beat Frequency (Hz)
1	8
2	9
3	9.5
Control subjects	Nasal Ciliary Beat Frequency (Hz)
1	11.58
2	10
3	10
4	12
5	11.35
6	10.2
7	13
8	9
9	10.6
10	12.3
11	11
12	12
13	12.5
14	10.8
15	9.1
16	14
17	10.7
18	9.5
19	9.5
20	11.8
21	11
22	9.5
23	9
24	10.8
25	14
26	9.75
27	9.75
28	11.75
29	10.5
30	10.5
Disease Group	Nasal ciliary beat frequency (Hz), mean ± SD
*MYO7A* (n = 5)	10.20±1.44
*CDH23* (n = 3)	8.33±0.76
USH1 (n = 8)	9.68±1.38
Control (n = 30)	10.88±1.36

We also evaluated whether ciliary beat frequency were different between *MYO7A* and *CDH23* patients. No statistical differences were found between *MYO7A* patients (10.20±0.66 Hz) and *CDH23* patients (8.83±0.44 Hz, Kruskal-Wallis test followed by Dunńs test). However, nasal ciliary beat frequency was significantly lower in *CDH23* patients than in controls (p<0.05, Kruskal-Wallis Test followed by Dunńs test) (Fig. 3B).

## Discussion

A high number of mutations responsible for USH have been identified by screenings performed in USH patients. The effect of variants that lead to premature stop codons is not questioned; however, it is complicated to predict the consequences of missense, silent and intronic changes, in order to discriminate neutral variants from those with pathogenic effect. Variants putative to affect the splicing process are usually considered pathogenic on the basis of their conservation in the canonical splice site, their absence in control samples, cosegregation with the disease in families and results from bioinformatic predictions [26].

Four bioinformatic programs (*NNSplice, SpliceView, HSF* and *NetGene2*) were used to predict the damaging effect of the variants identified in the USH1 genes of our cohort and eighteen changes were selected as putative to affect the splicing process (Table 4). These results are only computational predictions so, additional studies are necessary to confirm the effect on the mRNA processing. Minigenes and RNA assays were performed to achieve this goal.

Minigene assays revealed that only seven of the eighteen studied variants were affecting the splicing process (Table 5). The bioinformatic analysis of these seven variants had shown high scores. A maximum score of 4, for five of them: c.470G>A, c.1342_1343delAG and c.3652G>A in the *MYO7A* gene, c.2289+1G>A in *CDH23* gene and c.3717+2dupTT in *PCDH15* gene. The other two variants (c.6049G>A and c.8722+1delG in the *CDH23* gene) showed a score of 3. However, other studied variants that also reached a high score (≥3) did not show any effect in the splicing process when they were analyzed by minigenes. According to these results, all types of putative pathogenic variants should be analyzed by bioinformatic tools, and, when high scores are observed, minigene analysis should be performed to avoid false positive. See Table 5.

However, the splicing processes observed by minigenes would not necessarily reflect the real processing in affected tissues. In our study, we could analyze RNA from nasal epithelial cells from patients or carriers of eight variants previously analyzed by minigene constructions, to determine their effect on splicing process in patient’s tissues. Analysis of the RT-PCR amplification of nasal cells allowed us to confirm that four of the eight studied variants, one *MYO7A* (c.5856G>A) and three *CDH23* (c.2289+1G>A, c.6049G>A and c.8722+1delG), were affecting the splicing process. The same results were obtained by minigene constructions for the eight studied variants, except for two *CDH23* changes, c.2289+1G>A and c.6049G>A.

For the c.2289+1G>A variant the different results obtained by both methods are due to the minigene construction did not contain the entire intron 21 of the *CDH23* gene.

In the study of the c.6049G>A variant by analysis of RT-PCR amplification from nasal cells we were not able to amplify the mutant transcript. This fragment could be degraded by nonsense mediated decay mechanism (NMD), decreasing the mRNAs levels and therefore, the protein levels. However, by minigene construction we had observed in this case the skipping of exon 46.

In three cases (c.5856G>A (*MYO7A*), c.2289+1G>A (*CDH23*) and c.6049G>A (*CDH23*)), we could only amplify one of the two alleles due to the low USH1 genes expression level in this tissue. In fact, these USH transcripts are only detectable by nested PCR. Accordingly, it is advisable to detect the presence of heterozygous changes by sequencing, the studied mutation or other SNPs, to confirm the amplification of the two alleles and avoid incorrect conclusions when apparently only one fragment is obtained after PCR.

Therefore, minigenes are a good approach to ascertain the pathogenic nature of splice variants when is difficult to obtain RNA from patients’ tissues, as in the case of USH genes, that present a restricted expression profile associated with photoreceptors and inner hair cells. These constructions are expressed in living cells where the splicing machinery remains intact.

In addition, we can confirm that the analysis of nasal epithelial cells is an alternative method to discriminate neutral Usher variants from those with a pathogenic effect on the splicing process.

Finally, we have also demonstrated that the nasal ciliated epithelium of USH1 patients has a lower ciliary beat frequency than control subjects. Armengot et al. [12] suggested that mutations in the USH1 and USH2 genes could be responsible for the lower ciliary beat frequency. They observed a low beat frequency in USH2 patients. However, the ciliary activity was sufficient to operate normally and no clinical consequences were observed in these patients.
